# Clinical Immunogenicity Evaluation of Eptinezumab, a Therapeutic Humanized Monoclonal Antibody Targeting Calcitonin Gene-Related Peptide (CGRP) for the Preventive Treatment of Migraine

**DOI:** 10.3389/fimmu.2021.765822

**Published:** 2021-10-25

**Authors:** Susan Pederson, David M. Biondi, Brent Allan, Roger Cady, Barbara Schaeffler, Brian Baker, John Latham

**Affiliations:** ^1^ Lundbeck Seattle BioPharmaceuticals, Inc., Bothell, WA, United States; ^2^ Alder BioPharmaceuticals, Inc. (CKA Lundbeck Seattle BioPharmaceuticals, Inc.), Bothell, WA, United States

**Keywords:** eptinezumab, immunogenicity, anti-drug antibody, monoclonal antibody, migraine, neutralizing antibody, calcitonin gene-related peptide (CGRP)

## Abstract

**Background:**

Eptinezumab is a humanized monoclonal antibody that selectively binds calcitonin gene-related peptide and is indicated for the preventive treatment of migraine in adults. This analysis characterizes the immunogenic profile of eptinezumab using data from clinical trials of eptinezumab for migraine prevention.

**Methods:**

Immunogenicity data were collected from five studies that included 2076 patients with episodic or chronic migraine treated with eptinezumab at dose levels ranging from 10 to 1000 mg, administered intravenously for up to 4 doses at 12-week intervals. Anti-drug antibody (ADA) results were available from 2074 of these patients. Four studies were randomized, double-blind, placebo-controlled trials with ADA monitoring for up to 56 weeks; one was a 2-year, open-label, phase 3 safety study with ADA monitoring for 104 weeks. Patients who had a confirmed ADA-positive result at the end-of-study visit were monitored for up to 6 additional months. Development of ADA and neutralizing antibodies (NAbs) were evaluated to explore three key areas of potential impact: pharmacokinetic exposure profile (eptinezumab trough plasma concentrations), efficacy (change in monthly migraine days), and safety (rates of treatment-emergent adverse events). These studies included methods designed to capture the dynamics of a potential humoral immune response to eptinezumab treatment, and descriptive analyses were applied to interpret the relationship of ADA signals to drug exposure, efficacy, and safety.

**Results:**

Pooled across the five clinical trials, treatment-emergent ADAs and NAbs occurred in 15.8 and 6.2% of eptinezumab-treated patients, respectively. Highly consistent profiles were observed across all studies, with initial onset of detectable ADA observed at the week 8 measurement and maximal ADA frequency and titer observed at week 24, regardless of eptinezumab dose level or number of doses. After 24 weeks, the ADA and NAb titers steadily declined despite additional doses of eptinezumab.

**Interpretation:**

Collectively, these integrated analyses did not demonstrate any clinically meaningful impact from ADA occurring after treatment with eptinezumab. The ADA profiles were low titer and transient, with the incidence and magnitude of ADA or NAb responses declining after week 24. Development of ADAs and NAbs did not impact the efficacy and safety profiles of eptinezumab.

## Introduction

Eptinezumab, a calcitonin gene-related peptide (CGRP) antagonist indicated for the preventive treatment of migraine in adults (1), is a humanized immunoglobulin G1 (IgG1) monoclonal antibody (mAb) that binds CGRP with high affinity (2). It has demonstrated migraine preventive effects across the spectrum of migraine attack frequency, with statistically significant reduction from baseline in migraine frequency established as early as 1 day after the initial infusion and sustained throughout the 12-week dosing interval, with an acceptable safety and tolerability profile (3–11).

Like almost all biological proteins, mAbs have the potential to trigger an immune response after administration (12–16). In most cases, associated immunogenicity is multifactorial, with intrinsic factors, systems biology, conditions of use, patient-related factors, and product quality all potentially contributing. As with other humanized and fully recombinant human IgG1 mAbs (13), the non-human germline sequences in the complementarity-determining regions (CDRs) are the structural features of eptinezumab with the greatest immunogenic potential. Eptinezumab contains a human IgG1 Fc region sequence that corresponds to the two most common allotypes, G1m1,17 and G1m3, with the single exception of position 297, which was changed from an asparagine to an alanine to prevent N-linked glycosylation of the heavy chain. The CDR sequences are derived from a parental rabbit anti-alpha CGRP antibody; the six CDR sequences from the original rabbit antibody VL and VH regions were grafted into human VL and VH framework sequences that were most similar to the rabbit framework sequence. To retain full affinity of the humanized antibody for CGRP, several amino acids in the human framework were substituted with the corresponding rabbit amino acid to preserve antigen-binding affinity. The remainder of the variable regions represent a human germ-line sequence.

The clinical development of eptinezumab included assessments of immunogenicity. Comprehensive evaluations were performed to detect and characterize immune responses observed within the studies and to identify any correlation of immunogenicity with pharmacokinetic (PK), pharmacodynamic, efficacy, and safety endpoints. The objective of the integrated summary of immunogenicity analysis was to pool immunogenicity data from the five clinical studies of eptinezumab conducted in patients with migraine in order to robustly characterize the immunogenic profile of eptinezumab within this population.

## Materials and Methods

### Study Designs

The clinical development program of eptinezumab in migraine prevention included four randomized, double-blind, placebo-controlled trials and one open-label safety study (**Table 1**). Approval for each study was provided by the independent ethics committee or institutional review board of the study sites. All studies were conducted in accordance with Good Clinical Practice guidelines, the principles of the Declaration of Helsinki, and all applicable regulatory requirements. Patients provided written informed consent prior to initiation of any study procedures. Each study is registered on *ClinicalTrials.gov* (NCT01772524, NCT02275117, NCT02559895, NCT02974153, NCT02985398).

**Table 1:** Overview of clinical studies contributing to the immunogenicity evaluation for eptinezumab.

Study	Description (*ClinicalTrials.gov* ID)	Migraine diagnosis	Dose levels Number of doses (schedule)	Epti-treated patients with ADA results
Study 1	Phase 1b, DB/R/PC (NCT01772524) (3)	EM	1000 mg, placebo Single dose (day 0)	1000 mg: 81
Study 2	Phase 2, DB/R/PC (NCT02275117) (4)	CM	10, 30, 100, 300 mg, placebo Single dose (day 0)	300 mg: 120 100 mg: 122 30 mg: 122 10 mg: 129
PROMISE-1	Phase 3, DB/R/PC (NCT02559895) (5, 17)	EM	30, 100, 300 mg, placebo Four doses (day 0, weeks 12, 24, 36)	300 mg: 224 100 mg: 223 30 mg: 219
PROMISE-2	Phase 3, DB/R/PC (NCT02974153) (7, 8)	CM	100, 300 mg, placebo Two doses (day 0, week 12)	300 mg: 350 100 mg: 356
PREVAIL	Open-label* (NCT02985398) (9)	CM	300 mg Eight doses* (day 0, weeks 12, 24, 36, 48, 60, 72, 84)	300 mg: 128
			Total	2074

*In analyses included in this paper, data from only the first 4 doses of PREVAIL are included due to the fact that PREVAIL was ongoing and the interim analysis of the primary treatment phase (first 4 doses) was planned for inclusion in these analyses. ADA, anti-drug antibody; CM, chronic migraine; DB/R/PC, double-blind, randomized, placebo-controlled; EM, episodic migraine; Epti, eptinezumab.

Two of the placebo-controlled trials were single-dose studies: one in patients with episodic migraine [study 1 (3)] and one in patients with chronic migraine [study 2 (4)]. The remaining three trials were multiple-dose studies: PROMISE-1 evaluated eptinezumab for up to 4 doses (1 year) in patients with episodic migraine (5, 17), PROMISE-2 evaluated eptinezumab for up to 2 doses (6 months) in patients with chronic migraine (7, 8), and PREVAIL evaluated eptinezumab for up to 8 doses (2 years) in patients with chronic migraine (9). Though PREVAIL included 8 doses, the integrated analyses herein only include the interim study data (i.e., the primary treatment phase, or first 4 doses), as the study was still ongoing when the integrated summary of immunogenicity report was finalized for submission of the biological licensing application. In all trials, study drug was administered by intravenous infusion lasting 30 minutes (PROMISE-2 and PREVAIL) or 1 hour (study 1, study 2, and PROMISE-1).

### Assessment of Anti-Drug Antibodies

Immunogenicity sampling time points are shown in **Supplemental Table 1**. In each study, samples were collected prior to study drug administration on day 0 and regularly throughout each study at similar time points for analysis. Three of the studies included a 2-week time point to evaluate early seroconversion, followed by sampling at 4-week intervals to the end of study (EOS) and with all studies accounting for the half-life of eptinezumab [27 days (18)] by collecting samples at least 20 weeks (5 half-lives) after the last administration. The scheduled duration of anti-drug antibody (ADA) monitoring for the placebo-controlled studies and primary treatment phase of PREVAIL extended to 56 weeks, and for the PREVAIL secondary treatment phase extended to 104 weeks. Patients who tested positive for ADAs at the time of the last study visit were asked to provide up to two additional blood samples for immunogenicity testing at 3-month intervals to evaluate transient vs persistent ADA responses.

Immunogenicity was assessed in serum from all patients who received eptinezumab, using first- and second-generation binding antibody assays that were validated according to industry and regulatory recommendations (15, 16, 19–22). The first-generation assay supported study 1; the second-generation assay supported the remaining four studies and is summarized here. Validation and in-study performance data demonstrated the methods were suitable for their intended use and provided a scientifically sound framework for the immunogenicity assessment of eptinezumab.

The second-generation ADA assay was formatted as a homogeneous electrochemiluminescence (ECL) bridging assay. A sample pre-treatment step was included to improve sensitivity in the presence of residual drug and resulted in a drug-tolerant method for detection of eptinezumab-reactive ADAs of IgG, IgM, and IgA isotypes. Test samples were diluted 1:50 and, based on a surrogate positive control, the relative sensitivity was determined to be 87.34 ng/mL and 67.99 ng/mL for the screening and confirmatory steps of the method, respectively. Inter-assay precision showed a percent coefficient of variability (%CV) of 9.50%, with drug tolerances of >1000 µg/mL, >100 µg/mL, 25 µg/mL, and 25 µg/mL for positive controls in the high-control set at 2500 ng/mL, mid-control set at 250 ng/mL, screening low-control set at 110 ng/mL, and confirmatory low-control set at 85 ng/mL, respectively.

Sample analysis was performed using a tiered approach that consisted of a screening tier to detect ADAs, a confirmatory tier to ensure detected ADAs were specific to the drug, and a third tier to serve as a semi-quantitative measurement of the amount of antibody (titer) that was present. A fourth tier was also included as a specificity assessment to characterize the epitope binding of the response to distinguish ADAs that were reactive to the antibody framework from ADAs that were reactive with the CDRs.

To complete the immunogenicity testing paradigm, a neutralizing antibody (NAb) assay was developed to characterize confirmed ADAs for neutralizing potential. The NAb assay was a qualitative ligand-binding assay, formatted as competitive inhibition method, that evaluated the ability of anti-eptinezumab antibodies to block binding of the drug to the target ligand, human α-CGRP. The dynamics of the humoral immune response to eptinezumab using this tiered approach were described in terms of ADA positive vs negative status, ADA titer, and NAb positive vs negative status.

### Assessment of Pharmacokinetic Exposure, Efficacy, and Safety

Blood plasma samples for pharmacokinetic evaluation in eptinezumab-treated patients were collected at the same time points outlined in **Supplemental Table 1**. Trough plasma concentration (C_trough_) values were based on the sample taken just prior to the next dose administration for treatment visits and at each scheduled visit when available for the PK population. The plasma concentration values used represent all analytically valid plasma concentration results reported.

In all studies, patients recorded migraine and headache data daily using an eDiary. The efficacy outcome used for this analysis was defined as the change from baseline in monthly migraine days (MMDs).

Adverse events were collected from the time of informed consent through the final patient visit. Verbatim descriptions of adverse events were mapped to the Medical Dictionary for Regulatory Activities (MedDRA) thesaurus terms and converted to the same version (version 20.1) for all studies. A treatment-emergent adverse event (TEAE) was defined as an adverse event with a start date and time on or after the date and time of first dose of study drug administration. For the current analysis, impact on the incidence of TEAEs and adverse events of special interest (AESIs) was evaluated. AESIs are listed in **Supplemental Table 2**.

### Statistical Analysis

Analysis populations included the safety population (all patients who received eptinezumab; summarized by the treatment they received), the full analysis population (all patients who received treatment; summarized by the treatment to which they were randomized), and the PK population (all patients in the safety population who also had the PK parameters required for the analysis).

For this analysis, summary statistics, including the number of patients (n), mean, standard deviation, median, minimum, and maximum were evaluated for continuous variables. Generally, the minimum and maximum values were presented to the same decimal precision as the raw values, the mean and median values to one more, and the standard deviation to two more decimal places than the raw values. For categorical variables, per category, the absolute counts (n) and percentages (%) of patients with data and, if appropriate, the number of patients with missing data, were presented. Percentages are presented to one decimal place.

Details of the missing data rules used for the individual studies are used for these analyses with the exception of the migraine data for study 1, which was re-calculated to match the other studies. A brief summary of these rules is provided here with additional details reported in the respective study publications. For TEAE, PK, ADA, and NAb data, no missing data imputation was used. For the analysis of MMDs, missing data were imputed using either normalization or a weighted estimate. If migraine day data were missing for ≤7 days in a 28-day interval, the results were normalized to 28 days by multiplying the observed results by the inverse of the completion rate. If migraine day data were missing for >7 days in a 28-day interval, a weighted estimate based upon the results of the current and previous months was used, with greater weight being applied to the current month as the completion rate increased. For the pivotal phase 3 studies (PROMISE-1 and PROMISE-2) MMD change from baseline, analysis of covariance (ANCOVA) was performed to estimate the mean, the mean difference from ADA-negative, 95% confidence interval (95%CI), and p-value for the ADA-positive subpopulation.

ADA incidence data from study 1 were excluded from the pooled analysis due to the different assessment methodology (first-generation vs second-generation assay) used. The anti-eptinezumab antibody titer values were not pooled across the studies to evaluate titer by indication; results were summarized separately for each study.

## Results

### ADA/NAb Response Dynamics

**Figure 1:**
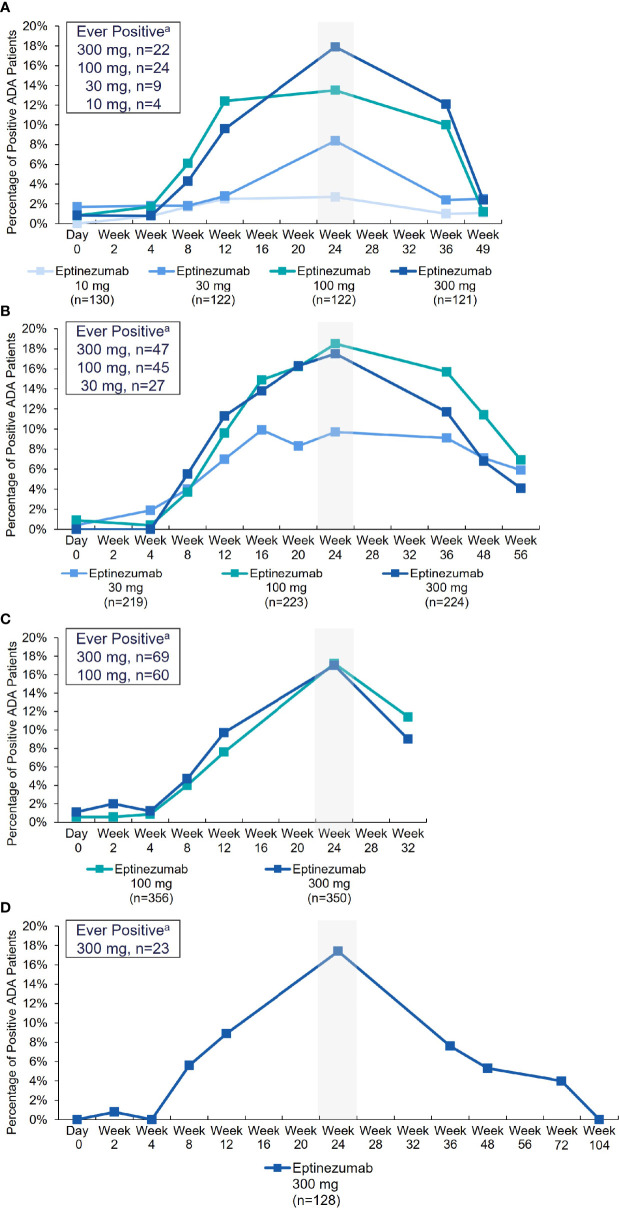
Percentage of ADA-positive patients by visit and treatment (safety population) in: **(A)** Study 2, **(B)** PROMISE-1, **(C)** PROMISE-2, and **(D)** PREVAIL. ^a^Represents number of patients ever ADA positive over the entire time course analyzed for each study. Note: The denominator for the percentage is the number of patients with ADA sampling results per treatment group at each visit. ADA, anti-drug antibody.

The incidence of treatment-emergent ADA across study 2, PROMISE-1, PROMISE-2, and PREVAIL was 15.9% (316/1993), with 6.2% (124/1993) of subjects with ADA results exhibiting antibodies with neutralizing potential (**Table 2**). The time course of ADA/NAb development was highly consistent across all four clinical studies (**Figure 1**), with initial onset of positivity observed at the week 8 measurement and maximal ADA-positive frequency at week 24, regardless of eptinezumab dose level or number of doses. A total of 11 (13.6%) patients who received a single 1000-mg dose in study 1 had treatment-emergent anti-eptinezumab immunoreactivity results (first-generation assay); 4 (4.9%) had confirmed NAb.

**Table 2:** Incidence of ADA/NAb by study (safety population)*.

	Phase 2		Phase 3			All studies
	Study 1 (3)*	Study 2 (4)	PROMISE-1 (5, 17)	PROMISE-2 (7, 8)	PREVAIL (9)	
Eptinezumab-treated patients with ADA results, N	81	493	666	706	128	2074
ADA positive at any time point, n (%)^†^	11 (13.6)	59 (12.0)	119 (17.9)	129 (18.3)	22 (17.2)	340 (16.4)
Treatment-emergent ADA, n (%)	11 (13.6)	55 (11.2)	116 (17.4)	123 (17.4)	22 (17.2)	327 (15.8)
NAb positive at any time point, n (%)^‡^	4 (4.9)	18 (3.7)	52 (7.8)	45 (6.4)	9 (7.0)	128 (6.2)

*Study 1 utilized first-generation assay methodology. All other studies utilized second-generation assay methodology, and precautions are necessary when comparing results. ^†^(Number of ADA-positive patients/Total number of eptinezumab-treated patients with ADA results) × 100. ^‡^(Number of NAb-positive patients/Total number of eptinezumab-treated patients with ADA results) × 100. ADA, anti-drug antibody; NAb, neutralizing antibody.

**Figure 2:**
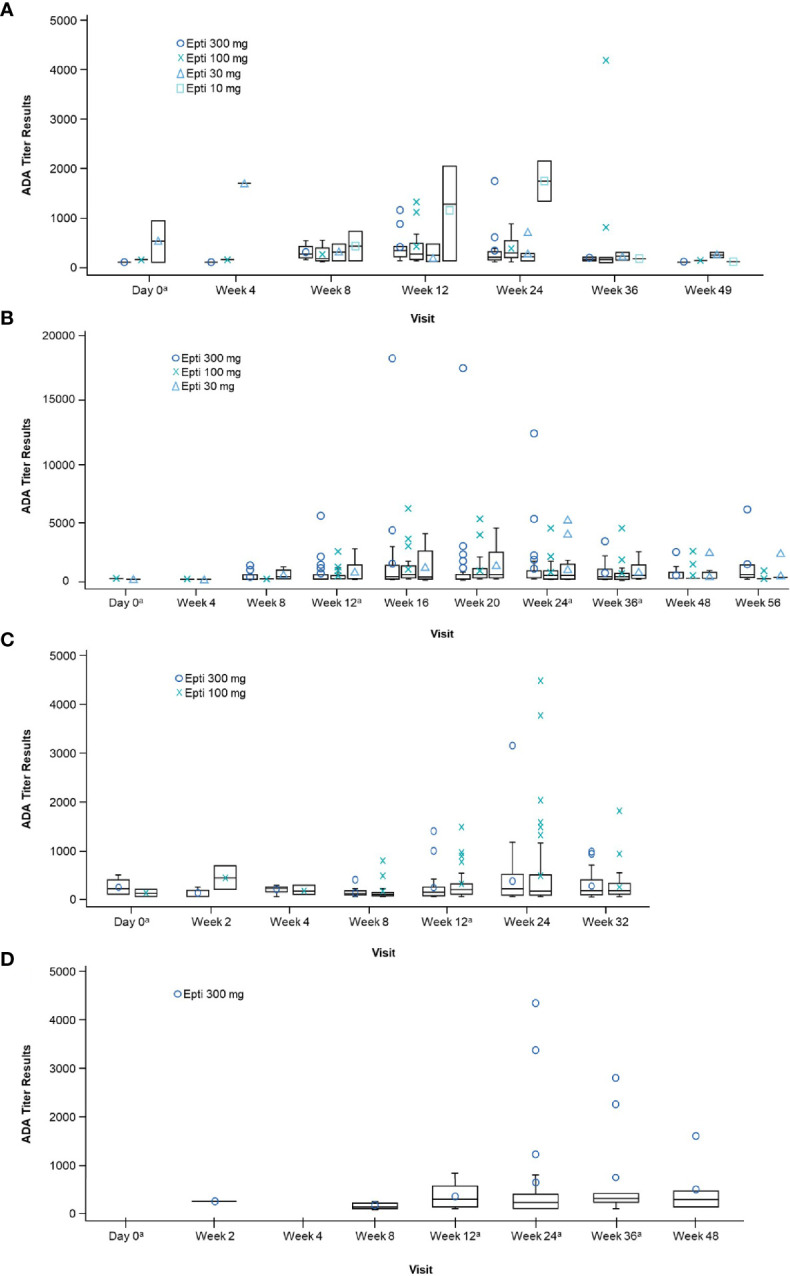
Boxplot of positive ADA titer results by visit and treatment (safety population) in: **(A)** Study 2, **(B)** PROMISE-1, **(C)** PROMISE-2, and **(D)** PREVAIL. ^a^On visits that coincided with treatment, ADA was measured pre-dosing. Note: Only patients with a confirmed ADA-positive status are summarized at each visit. ADA, anti-drug antibody; Epti, eptinezumab. ADA titer refers to the value obtained within screening assay through a series of dilutions beginning with the minimal required dilution of confirmed positive samples.

The time-course of evolution of ADA titer by treatment group across study 2, PROMISE-1, PROMISE-2, and PREVAIL are illustrated in **Figure 2**. In all studies, mean ADA titer was maximal at the week 24 measurement, declining thereafter; there was no clear dose-response relationship between the distribution of ADA titer values and the eptinezumab dose administered.

The fourth tier of the ADA assay, the specificity tier, represents a competitive inhibition format of the ADA screening assay, using ALD306, a Pichia-expressed monoclonal antibody that has the same framework and similar glycosylation profile as ALD403 (eptinezumab), but different CDRs, and CHO-derived ALD403, an antibody molecule produced in CHO cells with the same CDRs as Pichia-derived ALD403 (eptinezumab). Confirmed ADA positive samples were characterized for specificity. The aggregated results for all sample timepoints were summarized for each study where the signals were mainly reactive with the CHO-expressed eptinezumab, demonstrating specificity for the CDRs of eptinezumab rather than the IgG1 framework region. The Pichia-expressed human IgG1 mAb containing the unrelated CDR sequence (ALD306) showed negligible inhibition of the signal. **Tables 3** and **4** present the Specificity data from the PROMISE 1 and PROMISE 2 studies, respectively.

**Table 3:** Specificity of ADA signals detected at all timepoints in PROMISE 1.

Statistic	Eptinezumab dose			Overall N=666
	300 mg N=224	100 mg N=223	30 mg N=219	
Number of confirmed ADA-positive results for all timepoints	176	188	118	482
Reactive with CHO ALD403 only, n (%)	130 (73.9)	137 (72.9)	84 (71.2)	351 (72.8)
Reactive with ALD306 only, n (%)	5 (2.8)	5 (2.7)	6 (5.1)	16 (3.3)
Reactive with CHO ALD403 + ALD306, n (%)	22 (12.5)	25 (13.3)	11 (9.3)	58 (12.0)
Not reactive with either CHO ALD403 or ALD306, n (%)	19 (10.8)	21 (11.2)	17 (14.4)	57 (11.8)

ADA, anti-drug antibody; CHO, Chinese hamster ovary expressed antibody.

**Table 4:** Specificity of ADA signals detected in study eptinezumab PROMISE 2 Study (all timepoints).

Statistic	Eptinezumab dose		Overall N=706
	300 mg N=350	100 mg N=356	
Number of confirmed ADA-positive results for all timepoints	145	138	283
Reactive with CHO Eptinezumab only, n (%)	100 (69.0)	97 (70.3)	197 (69.6)
Reactive with ALD306 only, n (%)	14 (9.7)	10 (7.2)	24 (8.5)
Reactive with CHO Eptinezumab + ALD306, n (%)	10 (6.9)	23 (16.7)	33 (11.7)
Not reactive with either CHO Eptinezumab or ALD306, n (%)	23 (15.9)	11 (8.0)	34 (12.0)

ADA, anti-drug antibody; CHO, Chinese hamster ovary cell expressed antibody.

### Relationship to Drug Exposure

Within each study, relationships between ADA or NAb status and mean eptinezumab plasma concentrations were evaluated at C_trough_, the most sensitive area of the exposure profile. In both pivotal phase 3 studies (PROMISE-1 and PROMISE-2), mean C_trough_ concentrations were lower for the ADA- and NAb-positive subpopulations than for the ADA- and NAb-negative subpopulations at all evaluated doses and timepoints. At the week 24 visit, where the maximal incidence and titer of the ADA response was observed, mean C_trough_ plasma concentrations in the ADA-positive subgroups were 49.0 to 61.5% compared to that of the ADA-negative subgroups for eptinezumab 100 mg and 67.0 to 81.1% compared to that of the ADA-negative subgroups for eptinezumab 300 mg in PROMISE-1 and PROMISE-2, respectively.

In spite of the decrease observed in the mean C_trough_ plasma concentrations in the ADA-positive subgroups in both studies, mean C_trough_ levels at week 24 following eptinezumab 100 mg or 300 mg remained above established plasma concentrations that support the 90% maximal efficacy (EC_90_) thresholds (18). In PROMISE-2, mean C_trough_ levels at week 24 were ≥6% above the established EC_90_ for CM patients (0.98 μg/mL), at 3.47 μg/mL and 10.18 μg/mL in the eptinezumab 100- and 300-mg groups, respectively. Similar observations were found in the PROMISE-1 study, where the drug levels remained above the EC_90_ for EM patients (1.65 μg/mL), even among the highest ADA titers. These data support the observed absence of a negative influence on efficacy with a conclusion that ADA-positive status is not associated with a clinically meaningful change in the extent of drug exposure following the intravenous administration of eptinezumab 100 or 300 mg every 12 weeks.

### Relationship to Efficacy

**Figure 3:**
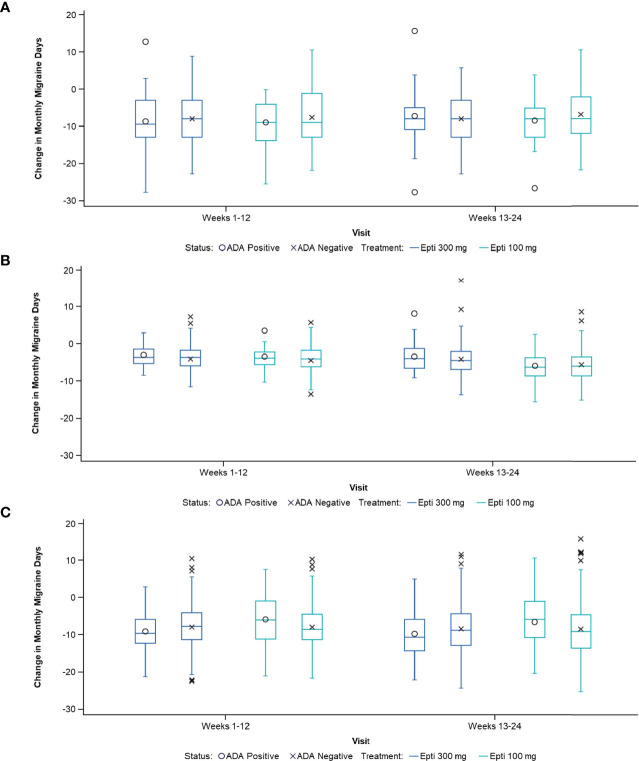
Change in MMDs by ADA status in: **(A)** Study 2, **(B)** PROMISE-1, and **(C)** PROMISE-2. ADA, anti-drug antibody; Epti, eptinezumab, MMDs, monthly migraine days.

Despite the presence of ADA or NAb, the migraine preventive efficacy of eptinezumab was maintained. The change in MMDs by ADA status was remarkably consistent for ADA-positive vs ADA-negative patients in the three studies in which this relationship was evaluated (Study 2, PROMISE 1, and PROMISE 2; **Figure 3**). Similar results were obtained for the NAb-positive vs NAb-negative subpopulations (**Table 5**). Additionally, ADA titer had no apparent impact on efficacy in the 100- or 300-mg eptinezumab treatment groups, indicating that increased ADA titer did not result in a decrease in the eptinezumab efficacy.

**Table 5:** Change in monthly migraine days by ADA and NAb status in Study 2, PROMISE-1, and PROMISE-2 (full analysis population).

	Study 2							
	Eptinezumab 300 mg				Eptinezumab 100 mg			
	ADA-positive	ADA-negative	NAb-positive	NAb-negative	ADA-positive	ADA-negative	NAb-positive	NAb-negative
Number of patients, n	22	91	7	15	23	95	8	14
Weeks 1–12 (SD)	-8.5 (8.40)	-8.1 (6.71)	-9.7 (10.73)	-8.0 (7.45)	-9.0 (6.23)	-7.3 (7.01)	-8.8 (4.74)	-8.8 (7.18)
Weeks 13–24 (SD)	-7.5 (8.75)	-8.3 (6.51)	-9.0 (10.49)	-6.8 (8.13)	-8.4 (7.00)	-6.8 (6.89)	-7.8 (6.11)	-8.4 (7.75)
	**PROMISE-1**							
Number of patients, n	46	176	16	30	46	175	22	24
Weeks 1–12 (SD)	-3.9 (4.79)	-4.4 (3.56)	-3.6 (3.37)	-4.0 (2.48)	-4.3 (2.56)	-3.8 (3.38)	-4.1 (2.48)	-4.5 (2.66)
Weeks 13–24 (SD)	-4.2 (3.85)	-5.0 (4.02)	-3.7 (4.67)	-4.5 (3.40)	-4.8 (3.32)	-4.6 (3.56)	-4.7 (3.51)	-4.8 (3.21)
	**PROMISE-2**							
Number of patients, n	69	281	19	49	60	296	26	33
Weeks 1–12 (SD)	-9.3 (5.95)	-8.1 (5.95)	-8.4 (7.09)	-9.7 (5.55)	-6.1 (6.81)	-8.1 (5.85)	-6.7 (7.00)	-5.6 (6.79)
Weeks 13–24 (SD)	-10.1 (6.75)	-8.7 (6.70)	-9.9 (7.19)	-10.1 (6.72)	-6.6 (6.89)	-8.6 (7.02)	-7.0 (8.08)	-6.1 (5.90)

ADA, anti-drug antibody; NAb, neutralizing antibodies.

The ANCOVA analysis of data from the full analysis population (i.e., all patients treated with eptinezumab, including 30, 100, and 300 mg) showed a highly comparable reduction in frequency of migraine days for the weeks 1–12 and weeks 13–24 treatment periods (**Table 6**), as well for the overall weeks 1–24 treatment period, for the ADA-positive vs ADA-negative subpopulations. There was no apparent relationship between efficacy (reduction in frequency of MMDs for weeks 1–24) with ADA-positive status, ADA titer, or NAb-positive status for the 100-mg or 300-mg treatment groups.

**Table 6:** Change in monthly migraine days by ADA status in PROMISE-1 and PROMISE-2 (full analysis population; all dose levels combined)*.

	PROMISE-1		PROMISE-2	
	ADA-positive (n=119)	ADA-negative (n=599)	ADA-positive (n=129)	ADA-negative (n=577)
Weeks 1–12, mean change from baseline	–4.4	–4.3	–7.7	–8.0
Mean difference from ADA-negative (95%CI)	–0.08 (–0.70, 0.54)		0.29 (–0.83, 1.42)	
P-value	0.8022		0.6081	
Weeks 13–24, mean change from baseline	–4.9	–4.9	–8.4	–8.6
Mean difference from ADA-negative (95%CI)	0.08 (–0.62, 0.78)		0.27 (–1.02, 1.55)	
P-value	0.8201		0.6827	
Weeks 1–24, mean change from baseline	–4.6	–4.6	–8.1	–8.3
Mean difference from ADA-negative (95%CI)	0.00 (–0.62, 0.62)		0.28 (–0.87, 1.43)	
P-value	0.9972		0.6306	

*The estimated mean, mean difference from ADA negative, 95% CI, and p-value are from a post hoc analysis of covariance (ANCOVA), with the change from baseline measure as the response variable and overall ADA status and variables measuring the individual study stratification factors (baseline migraine days and prophylactic medication use) as the independent variables. ADA, anti-drug antibody; 95%CI, 95% confidence interval.

### Relationship to Safety

Across the five studies, the incidences of TEAEs were in general similar in ADA ever-positive patients (ever-positive meaning a confirmed positive result at any single visit) and ADA-negative patients, and no clear differences were seen between the eptinezumab doses. Similarly, there were no clear differences of AESI incidences between ADA ever-positive patients and ADA-negative patients (**Tables 7** and **8**).

**Table 7:** Summary of TEAEs and AESIs by anti-eptinezumab antibody (ADA) status for PROMISE-1.

Treatment	Eptinezumab 300 mg		Eptinezumab 100 mg		Eptinezumab 30 mg		Placebo (n=222)
	Ever positive (n=47)	Negative (n=177)	Ever positive (n=45)	Negative (n=178)	Ever positive (n=27)	Negative (n=192)	
Number of patients with any TEAE (%)	31 (66.0%)	98 (55.4%)	26 (57.8%)	115 (64.6)	16 (59.3%)	112 (58.3%)	132 (59.5%)
Number of patients with any AESI (%)	8 (17.0%)	14 (7.9%)	5 (11.1%)	18 (10.1%)	0	22 (11.5%)	11 (5.0%)

AESI, adverse event of special interest; TEAE, treatment-emergent adverse event. Ever positive represents a confirmed positive result at any single visit.

**Table 8:** Summary of TEAEs and AESI by anti-eptinezumab antibody (ADA) status for PROMISE-2.

Treatment group	Eptinezumab 300 mg		Eptinezumab 100 mg		Placebo (n=366)
	Ever positive (n=69)	Negative (n=281)	Ever positive (n=60)	Negative (n=296)	
Number of patients with any TEAE, n (%)	33 (47.8%)	149 (53.0%)	31 (51.7%)	124 (41.9%)	171 (46.7%)
Number of patients with any AESI, n (%)	5 (7.2%)	30 (10.7%)	7 (11.7%)	16 (5.4%)	18 (4.9%)

AESI, adverse event of special interest; TEAE, treatment-emergent adverse event. Ever positive represents a confirmed positive result at any single visit.

There were no cases of anaphylaxis or severe hypersensitivity reactions observed in study 1, study 2, PROMISE-1, or PROMISE-2. There was 1 serious treatment-emergent adverse event of special interest of anaphylactic reaction of Grade 2 severity reported in PREVAIL, where the serious criteria of medically important was reported by the investigator. The Sponsor assessed the event using clinical diagnostic criteria as outlined by the second symposium on the definition and management of anaphylaxis (23) and determined the adverse event may have been better described by the preferred term (PT) of allergic reaction under the system organ class (SOC) of immune system disorders (9).

Within the safety populations for study 2, PROMISE-1, PROMISE-2, and PREVAIL, 24/1995 (1.2%) eptinezumab-treated patients experienced mild or moderate adverse events that were coded to the PT of hypersensitivity. There was no apparent relationship to pre-existing or treatment-emergent ADA- or NAb-positive status or ADA titer category.

## Discussion

In this integrated analysis of data from five clinical studies of eptinezumab for the preventive treatment of migraine, the overall incidences of treatment-emergent ADA (15.9%) and NAb (6.2%) were slightly higher than other anti-CGRP mAbs indicated as migraine preventives (24); however, the presence of ADA and NAb did not appear to influence treatment efficacy or safety. Efficacy for migraine prevention was comparable in ADA-positive and ADA-negative subpopulations. There were no cases of severe hypersensitivity reactions and only 1 serious AESI of anaphylactic reaction, which did not fulfill the clinical diagnostic criteria for anaphylactic reactions as outlined by the second symposium on the definition and management of anaphylaxis (23). There was no evidence for a risk of immune complex-related hypersensitivity, consistent with the relatively low ADA titers observed for all eptinezumab dose levels, and there was no relationship between the ADA or NAb status and the incidence or severity of other adverse events of special interest. As the incidence of treatment-emergent ADAs in these studies consistently peaked at week 24, it is likely that any potential negative effects of immunogenicity would have manifested by this timepoint. Thus, these findings reduce uncertainty about the possible impact of immunogenicity on the sustainability of the treatment response and the potential for treatment-related hypersensitivity reactions with long-term administration.

Overall, the ADA frequency and titer profiles indicate the maximum amplitude of the treatment-emergent ADA response is attained following the second of the 3-monthly dose administrations regardless of the eptinezumab dose level. After 24 weeks the ADA titer declined, consistent with the half-life of endogenous human IgG (21-25 days). The reason for this decline is likely due to a combination of moderate to low titer levels, minimal boosting, and a natural clearance mechanism. ADA would be expected to be eliminated after approximately 5 half-lives, which amounts to about 16 weeks. Based on these data, the dynamics (in terms of both frequency and magnitude) were highly comparable for the 100- and 300-mg eptinezumab treatment groups through the complete time-course for each clinical study. Characterization of the ADA responses showed reactivity was directed toward the primary amino acid sequence (CDRs) of eptinezumab, which is typical for most therapeutic mAbs (13).

Although the analysis of potential relationships between ADA/NAb and drug exposure revealed differences in C_trough_ plasma concentrations between ADA-positive and ADA-negative patients, it did not identify any associated clinical impact. Though a decrease in serum concentration was observed, the drug levels remained above EC_90_, even among the highest ADA titers, supporting the conclusion that ADA-positive status is not associated with a clinically meaningful change in the extent of drug exposure.

The molecular design of eptinezumab effectively minimized product-related factors known to be associated with increased immunogenicity among mAbs. As demonstrated in the specificity evaluation of ADA, the responses were mainly reactive with the CHO-expressed ALD403, which has the same CDRs as ALD403 but is produced in mammalian cells rather than yeast cells, resulting in differences in the IgG1 framework region, IgG1 Fc region, or *Pichia*-derived glycans. Using the CHO ALD403 antibody with the same amino acid sequence as eptinezumab, expressed from a different host expression system, enabled pinpointing of the response directly to the CDRs of eptinezumab. Thus treatment-emergent ADAs associated with eptinezumab administration are not likely to enhance responses toward subsequent treatment with other therapeutic mAb products.

The clinical studies included in this analysis were designed to capture the dynamics of a potential humoral immune response to eptinezumab treatment, and descriptive analyses were applied to interpret the relationship of ADA/NAb signals to drug exposure, efficacy, and safety. Limitations of this analysis include the clinical trial population, which does not reflect all patients with episodic or chronic migraine, and long-term effects, which were only reported up to 1 year here. Further studies in a broader population with migraine and longer-term studies could further elucidate the efficacy and safety of eptinezumab (25).

In conclusion, the results of this analysis suggest that the clinical benefits and safety of eptinezumab for the preventive treatment of migraine are not impacted by immunogenicity.

## Data Availability Statement

In accordance with EFPIA’s and PhRMA’s “Principles for Responsible Clinical Trial Data Sharing” guidelines, Lundbeck is committed to responsible sharing of clinical trial data in a manner that is consistent with safeguarding the privacy of patients, respecting the integrity of national regulatory systems, and protecting the intellectual property of the sponsor. The protection of intellectual property ensures continued research and innovation in the pharmaceutical industry. Deidentified data are available to those whose request has been reviewed and approved through an application submitted to https://www.lundbeck.com/global/our-science/clinical-data-sharing.

## Ethics Statement

Approval for each study was provided by the independent ethics committee or institutional review board of the study sites. All studies were conducted in accordance with Good Clinical Practice guidelines, the principles of the Declaration of Helsinki, and all applicable regulatory requirements. Patients provided written informed consent prior to initiation of any study procedures.

## Author Contributions

SP, DB, BA, RC, BS, BB, and JL contributed to conception and design of this work. SP and DB contributed to the acquisition, analysis, and interpretation of this work. SP, DB, BA, RC, BS, BB, and JL contributed to the drafting and revision of this work. SP, DB, BA, RC, BS, BB, and JL provided final approval of the version to be published. All authors agree to be accountable for the content of the work.

## Funding

This study was funded by H. Lundbeck A/S (Copenhagen, Denmark).

## Conflict of Interest

All authors were full-time employees of company H. Lundbeck A/S (formally known as Alder BioPharmaceuticals), or one of its subsidiary companies, at the time of study and during manuscript preparation. BS has patents: Eptinezumab in the acute treatment of Migraines pending, and Eptinezumab in the treatment of MOH pending. SP, BB, and RC are full-time employees of H. Lundbeck A/S or one of its subsidiary companies.

The authors declare that this study received funding from H. Lundbeck A/S. The funder had the following involvement with the study: design and conduct of the study; data collection, management, and analysis; and preparation, review, and approval of the manuscript for publication.

## Publisher’s Note

All claims expressed in this article are solely those of the authors and do not necessarily represent those of their affiliated organizations, or those of the publisher, the editors and the reviewers. Any product that may be evaluated in this article, or claim that may be made by its manufacturer, is not guaranteed or endorsed by the publisher.

## References

[1] VYEPTI [package insert]. Bothell, WA: Lundbeck Seattle BioPharmaceuticals, Inc; (2021).

[2] Baker B Schaeffler B Cady R Latham J Whitaker T Smith J. Rational Design of a Monoclonal Antibody (mAB) Inhibiting Calcitonin Gene-Related Peptide (CGRP), ALD403, Intended for the Prevention of Migraine (P2.155). Neurology (2017) 88.

[3] Dodick DW Goadsby PJ Silberstein SD Lipton RB Olesen J Ashina M. Safety and Efficacy of ALD403, an Antibody to Calcitonin Gene-Related Peptide, for the Prevention of Frequent Episodic Migraine: A Randomised, Double-Blind, Placebo-Controlled, Exploratory Phase 2 Trial. Lancet Neurol (2014) 13:1100–7. doi: 10.1016/S1474-4422(14)70209-1 25297013

[4] Dodick DW Lipton RB Silberstein S Goadsby PJ Biondi D Hirman J. Eptinezumab for Prevention of Chronic Migraine: A Randomized Phase 2b Clinical Trial. Cephalalgia (2019) 39:1075–85. doi: 10.1177/0333102419858355 31234642

[5] Ashina M Saper J Cady R Schaeffler B Biondi DM Hirman J. Eptinezumab in Episodic Migraine: A Randomized, Double-Blind, Placebo-Controlled Study (PROMISE-1). Cephalalgia (2020) 40:241–54. doi: 10.1177/0333102420905132 PMC7066477 32075406

[6] Smith TR Janelidze M Chakhava G Cady R Hirman J Allan B. Eptinezumab for the Prevention of Episodic Migraine: Sustained Effect Through 1 Year of Treatment in the PROMISE-1 Study. Clin Ther (2020) 42:2254–65.e3. doi: 10.1016/j.clinthera.2020.11.007 33250209

[7] Lipton RB Goadsby PJ Smith J Schaeffler BA Biondi DM Hirman J. Efficacy and Safety of Eptinezumab in Patients With Chronic Migraine. PROMISE-2. Neurology (2020) 94:e1365–77. doi: 10.1212/WNL.0000000000009169 PMC7274916 32209650

[8] Silberstein S Diamond M Hindiyeh NA Biondi DM Cady R Hirman J. Eptinezumab for the Prevention of Chronic Migraine: Efficacy and Safety Through 24 Weeks of Treatment in the Phase 3 PROMISE-2 (Prevention of Migraine via Intravenous ALD403 Safety and Efficacy–2) Study. J Headache Pain (2020) 21:120. doi: 10.1186/s10194-020-01186-3 33023473 PMC7539382

[9] Kudrow D Cady RK Allan B Pederson SM Hirman J Mehta LR. Long-Term Safety and Tolerability of Eptinezumab in Patients With Chronic Migraine: A 2-Year, Open-Label, Phase 3 Trial. BMC Neurol (2021) 21:126. doi: 10.1186/s12883-021-02123-w 33740902 PMC7977171

[10] Smith TR Spierings ELH Cady R Hirman J Schaeffler B Shen V. Safety and Tolerability of Eptinezumab in Patients With Migraine: A Pooled Analysis of 5 Clinical Trials. J Headache Pain (2021) 22:16. doi: 10.1186/s10194-021-01227-5 33781209 PMC8008612

[11] Yan Z Xue T Chen S Wu X Yang X Liu G. Different Dosage Regimens of Eptinezumab for the Treatment of Migraine: A Meta-Analysis From Randomized Controlled Trials. J Headache Pain (2021) 22:10. doi: 10.1186/s10194-021-01220-y 33676408 PMC7937260

[12] Rosenberg AS. Immunogenicity of Biological Therapeutics: A Hierarchy of Concerns. Dev Biol (Basel) (2003) 112:15–21. 12762500

[13] Harding FA Stickler MM Razo J Dubridge RB. The Immunogenicity of Humanized and Fully Human Antibodies: Residual Immunogenicity Resides in the CDR Regions. MAbs (2010) 2:256–65. doi: 10.4161/mabs.2.3.11641 PMC2881252 20400861

[14] Shankar G Arkin S Cocea L Devanarayan V Kirshner S Kromminga A. Assessment and Reporting of the Clinical Immunogenicity of Therapeutic Proteins and Peptides-Harmonized Terminology and Tactical Recommendations. AAPS J (2014) 16:658–73. doi: 10.1208/s12248-014-9599-2 PMC4070270 24764037

[15] FDA. Guidance for Industry: Immunogenicity Assessment for Therapeutic Protein Products (2014). Available at: https://www.fda.gov/media/85017/download (Accessed February 2, 2021).

[16] FDA. Guidance for Industry: Assay Development and Validation for Immunogenicity Testing of Therapeutic Protein Products (2016). Available at: https://www.fda.gov/media/77796/download (Accessed February 2, 2021).

[17] Smith TR Janelidze M Chakhava G Cady R Hirman J Allan B. Eptinezumab for the Prevention of Episodic Migraine: Sustained Effect Through 1 Year of Treatment in the PROMISE-1 Study. Clin Ther (2020) 42:2254–65.e3. doi: 10.1016/j.clinthera.2020.11.007 33250209

[18] Baker B Schaeffler B Beliveau M Rubets I Pederson S Trinh M. Population Pharmacokinetic and Exposure-Response Analysis of Eptinezumab in the Treatment of Episodic and Chronic Migraine. Pharmacol Res Perspect (2020) 8:e00567. doi: 10.1002/prp2.567 32155317 PMC7064329

[19] FDA. Guidance for Industry: Assay Development for Immunogenicity Testing of Therapeutic Proteins (2009) (Accessed February 2, 2021).

[20] FDA. Bioanalytical Method Validation: Guidance for Industry (2018). Available at: https://www.fda.gov/files/drugs/published/Bioanalytical-Method-Validation-Guidance-for-Industry.pdf (Accessed February 2, 2021).

[21] USP Chapter 1106. United States Pharmacopeia. Chapter 1106. Immunogenicity Assays – Design and Validation of Immunoassays to Detect Anti-Drug Antibodies (2012). Available at: http://www.usp.org (Accessed February 2, 2021).

[22] EMEA. 2017 Guideline on Immunogenicity Assessment of Therapeutic Proteins (EMEA/CHMP/BMWP/14327/2006 Rev 1), 18 May (2017). Available at: http://www.ema.europa.eu/docs/en_GB/document_library/Scientific_guideline/2017/06/WC500228861.pdf (Accessed February 2, 2021).

[23] Sampson HA Munoz-Furlong A Campbell RL Adkinson NF Jr Bock SA Branum A. Second Symposium on the Definition and Management of Anaphylaxis: Summary Report–Second National Institute of Allergy and Infectious Disease/Food Allergy and Anaphylaxis Network Symposium. J Allergy Clin Immunol (2006) 117:391–7. doi: 10.1016/j.jaci.2005.12.1303 16461139

[24] Cohen JM Ning X Kessler Y Rasamoelisolo M Campos VR Seminerio MJ. Immunogenicity of Biologic Therapies for Migraine: A Review of Current Evidence. J Headache Pain (2021) 22:3. doi: 10.1186/s10194-020-01211-5 33413094 PMC7791637

[25] Spuntarelli V Negro A Luciani M Bentivegna E Martelletti P. Eptinezumab for the Treatment of Migraine. Expert Opin Biol Ther (2021) 21:999–1011. doi: 10.1080/14712598.2021.1931678 34009094

