# Supplementary material for: Clinical Immunogenicity Evaluation of Eptinezumab, a Therapeutic Humanized Monoclonal Antibody Targeting Calcitonin Gene-Related Peptide (CGRP) for the Preventive Treatment of Migraine
Source: Front Immunol. 2021 Oct 25;12:765822. doi: 10.3389/fimmu.2021.765822 (PMC8573262; doi:10.3389/fimmu.2021.765822)
Supplement: Supplementary file 1 [file DataSheet_1.pdf]

Supplementary Material

Supplemental Table 1 ADA sampling time points for each study*

Visit (target day)†	Study 1	Study 2	PROMISE-1	PROMISE-2	PREVAIL‡
Day 0	Pre-dose	Pre-dose	Pre-dose	Pre-dose	Pre-dose
Week 2 (Day 14)	X			X	X
Week 4 (Day 28)		X	X	X	X
Week 8 (Day 56)		X	X	X	X
Week 12 (Day 84)	X	X	X	X	X
Week 16 (Day 112)			X		
Week 20 (Day 140)			X		
Week 24 (Day 168)	X	X	X	X	X
Week 32/36 (Day 224/238)		X	X	X	X
Week 48/49 (Day 336/343)		X	X		X
Week 56 (Day 392)			X		X
Week 72 (Day 504)					X
Week 104 (Day 728)					X

*Patients who tested positive for ADA at the time of the last study visit were asked to provide up to two additional blood samples for immunologic testing at 3-month intervals for up to 6 months. †Analysis windows ranged from ±1 to ±7 days across studies. ‡In analyses included in this paper, data from only the first 4 doses of PREVAIL are included due to the fact that PREVAIL was ongoing and the interim analysis of the primary treatment phase (first 4 doses) was planned for inclusion in these analyses. ADA, anti-drug antibody.

Supplemental Table 2 Adverse events of special interest

Category	System organ class	Preferred term(s)
Hypersensitivity and anaphylactic events	Immune system disorders	Hypersensitivity, Anaphylactic reaction, and Anaphylactoid reaction
Events associated with C-SSRS	Psychiatric disorders	Suicidal behavior, Suicidal ideation, Suicidal attempt, and Self-injurious behavior
Cardiovascular events	Cardiac disorders	Atrial fibrillation, Bradycardia, Chest pressure, Palpitations, Sinus bradycardia, Sinus tachycardia, and Tachycardia
	Investigations	Blood pressure increase, Blood pressure systolic increase, Elevated blood pressure, Heart rate increased, Heart rate decreased, Heart rate irregular, ECG abnormal, ECG Q wave abnormal, ECG QT interval abnormal, and ECG QT prolonged
	Nervous system disorders	Seizure and Syncope
	Vascular disorders	Flushing, Hot flush, Hypertension, Hypotension, and Ischemia
Hepatic events	Investigations	Alanine aminotransferase increased, Aspartate aminotransferase increased, Bilirubin increased, Hepatic enzyme increased, Liver function test abnormal, and Transaminases increased
Events associated with study drug infusion (occurring within 1 week of dosing)	Skin and subcutaneous tissue disorders	Dermatitis bullous, Pruritus, Pruritus generalized, Rash, Rash macular, Rash macular-papular, Rash papular, Rash pruritic, and Urticaria
	General disorders and administration site conditions	Infusion site erythema, Infusion site extravasation, Infusion site pain, Infusion site paresthesia, Infusion site pruritus, Infusion site rash, Infusion site reaction, and Infusion site swelling

C-SSRS, Columbia–Suicide Severity Rating Scale; ECG, electrocardiogram.
